# Adapting the Pore Size of Individual, 3D-Printed CPC Scaffolds in Maxillofacial Surgery

**DOI:** 10.3390/jcm10122654

**Published:** 2021-06-16

**Authors:** David Muallah, Philipp Sembdner, Stefan Holtzhausen, Heike Meissner, André Hutsky, Daniel Ellmann, Antje Assmann, Matthias C. Schulz, Günter Lauer, Lysann M. Kroschwald

**Affiliations:** 1 Department of Oral and Maxillofacial Surgery, Faculty of Medicine “Carl Gustav Carus”, Technische Universität Dresden, Fetscherstraße 74, 01307 Dresden, Germany; David.Muallah@uniklinikum-dresden.de (D.M.); Guenter.Lauer@uniklinikum-dresden.de (G.L.); 2 Department of Mechanical Engineering, Institute of Machine Elements and Machine Design, Technische Universität Dresden, 01062 Dresden, Germany; Philipp.Sembdner@tu-dresden.de (P.S.); stefan.holtzhausen@tu-dresden.de (S.H.); 3 Department of Prosthetic Dentistry, University Hospital “Carl Gustav Carus”, Technische Universität Dresden, Fetscherstraße 74, 01307 Dresden, Germany; Heike.Meissner@uniklinikum-dresden.de; 4 Organical CAD/CAM, Ruwersteig 43, 12681 Berlin, Germany; Andre.Hutsky@organical-cadcam.com (A.H.); Daniel.Ellmann@organical-cadcam.com (D.E.); 5 Zahntechnik Schönberg, Altseidnitz 19, 01277 Dresden, Germany; Kontakt@Zahntechnik-Schoenberg.de; 6 Department of Oral and Maxillofacial Surgery, University Hospital Tübingen, Eberhard Karls Universität Tübingen, Osianderstraße 2-8, 72076 Tübingen, Germany; Matthias.Schulz@med.uni-tuebingen.de; 7 Centre for Translational Bone, Joint and Soft Tissue Research, University Hospital “Carl Gustav Carus”, Technische Universität Dresden, Fetscherstraße 74, 01307 Dresden, Germany

**Keywords:** calcium phosphate cement, pore size, augmentation, additive manufacturing

## Abstract

Three dimensional (3D) printing allows additive manufacturing of patient specific scaffolds with varying pore size and geometry. Such porous scaffolds, made of 3D-printable bone-like calcium phosphate cement (CPC), are suitable for bone augmentation due to their benefit for osteogenesis. Their pores allow blood-, bone- and stem cells to migrate, colonize and finally integrate into the adjacent tissue. Furthermore, the pore size affects the scaffold’s stability. Since scaffolds in maxillofacial surgery have to withstand high forces within the jaw, adequate mechanical properties are of high clinical importance. Although many studies have investigated CPC for bone augmentation, the ideal porosity for specific indications has not been defined yet. We investigated 3D printed CPC cubes with increasing pore sizes and different printing orientations regarding cell migration and mechanical properties in comparison to commercially available bone substitutes. Furthermore, by investigating clinical cases, the scaffolds’ designs were adapted to resemble the in vivo conditions as accurately as possible. Our findings suggest that the pore size of CPC scaffolds for bone augmentation in maxillofacial surgery necessarily needs to be adapted to the surgical site. Scaffolds for sites that are not exposed to high forces, such as the sinus floor, should be printed with a pore size of 750 µm to benefit from enhanced cell infiltration. In contrast, for areas exposed to high pressures, such as the lateral mandible, scaffolds should be manufactured with a pore size of 490 µm to guarantee adequate cell migration and in order to withstand the high forces during the chewing process.

## 1. Introduction

Maxillofacial surgeons are often challenged by complex bone defects caused by trauma, tumors, inflammation or long lasting edentulism. In many cases, the reconstruction of bony structures is necessary for the rehabilitation of the shape, function and aesthetics of the orofacial system. For this purpose, different materials, such as ready-made bone substitution materials (e.g., granulate, membranes or cones) or autologous bone, are used. An eligible bone substitute with outstanding properties is the autologous bone graft [1]. However, this requires harvesting from other anatomical sites, which is associated with donor site morbidity and limited capacity [2,3,4,5]. For this reason, the necessity to develop new synthetic bone substitute materials is increasing.

Three dimensional (3D) printing is an emerging technology in the medical field that offers new opportunities for tissue engineering and the reconstruction of bone [6,7,8]. Based on three-dimensional imaging, patient specific scaffolds can be manufactured additively. Different materials can be used for this purpose; one of them is calcium phosphate cement (CPC). CPC is a hydroxyapatite forming, synthetic bone substitution material, which mimics the inorganic part of human bone. Due to its material properties, such as its pasty consistency, biocompatibility and biodegradability, it has gained the attention, not only of scientists, but also of many clinicians [9,10,11]. CPC is osteoconductive, which means it is capable of guiding the growth and proliferation of osteoblasts on its surface. While the synthesis of other bio ceramics involves high temperature sintering, CPC can set and harden at room or body temperature at a nearly neutral pH. Its clinical benefit has been proven in several trials [12,13,14,15]. 

For successful bone reconstruction, the bone substitution material has to be permanently integrated into the defect site. This can be achieved by way of two mechanisms. The bone substitution material can be resorbed and replaced by the organism’s host bone [16]. Materials such as bovine collagen, or autologous as well as allogenic bone grafts, become integrated in this way [16,17]. The duration of this mechanism depends on the material’s biodegradability. For other materials, such as BioOss^®^ or CPC, the human body needs years to perform complete remodeling [18,19]. The functional integration of these materials is realized in a different way. Through integrated macro pores, blood-, bone- and stem cells infiltrate those scaffolds directly after implantation. This leads to the incorporation of the scaffold after a few months. Therefore, the pores in these bone substitution materials play a major role in a successful outcome. Bigger pore sizes, which may increase the infiltration of cells, come along with a smaller scaffold surface. The printing orientation also affects the surface area of the scaffolds. By printing the strands at a 45° angle to the scaffold’s edges, the surface can additionally be increased. A smaller surface could lead to decreased osteoconductivity of the scaffold. Additionally, wider pores and a smaller surface mean less stability. In maxillofacial surgery especially, the stability of bone scaffolds is crucial since high pressures emerge during the chewing process. 

Using 3D printing, the size of the pores can be adapted to a specific purpose. In regions such as the maxillary sinus, the scaffold’s stability might play a secondary role, whereas on the alveolar ridges, high stability is absolutely essential due to the high forces that emerge during the chewing process [20,21]. Considering these aspects, a defined pore size of CPC scaffolds for maxillofacial surgery could be of substantial clinical relevance. The optimal compromise between porosity, surface and stability needs to be determined. Many studies have investigated and approved CPC as a promising bone substitution material, but the ideal porosity of CPC scaffolds for specific indications has not yet been described [15,22,23]. This study aims to find the above mentioned optimal compromise between porosity, surface area and scaffold stability. We therefore investigated CPC scaffolds with six different porosities (0.1 mm, 0.23 mm, 0.36 mm, 0.49 mm, 0.62 mm and 0.75 mm) and two printing orientations (90° and 45°) in vitro, and compared the results with commercially available bone grafts. The scaffolds were colonized with human mesenchymal stem cells (hMSC) and were investigated regarding the depth of cell infiltration. Furthermore, the influence of strand arrangement and pore size on the scaffolds’ stability was studied. Moreover, in order to replicate the various in vivo conditions as accurately as possible, individual cone-beam computed tomography (CBCT) based CPC scaffolds presenting different parts of the maxillofacial region were printed and analyzed regarding their stability. In this regard, we hypothesized that an increasing pore size significantly influences not only migration but also the graft´s stability. Our results show the importance of the external and internal structure, especially for individual scaffolds in maxillofacial surgery.

## 2. Materials and Methods

### 2.1. Virtual Scaffold Planning

Cubic shaped and individual scaffolds were digitally planned. For individual scaffolds, geometries were designed based on patients’ cone beam data, in the manner of backward planning. First, the prosthetic restoration was set into the ideal position, determining the position needed for the dental implant. Based on the dental planning, boundary conditions were defined using cone-beam data (CBCT) for the individual scaffold to be designed. These boundary conditions represent geometric elements, such as planes or curves, that limit the dimension of the scaffold from a medical point of view and define the principal location of design features to be integrated (holes, cavities, etc.). Subsequently, the CPC scaffold was designed around the dental implant according to all clinical and geometric specifications. The use of patient data was approved by the local ethical review board (IRB00001473; file reference: EK1450420019).

### 2.2. Scaffold Fabrication

The scaffolds were fabricated from plottable CPC paste (INNOTERE Paste-CPC), manufactured by INNOTERE GmbH (Radebeul, Germany), by using a 3D plotting device (KOSY4, Elektronik and Mechanik GmbH, Thalheim, Germany), and were sterilized with γ-irradiation (25 kGy). For colonization studies, cubic-shaped scaffolds (10 × 10 × 10 mm) were plotted utilizing a 310 µm needle with a plotting speed of 8 mm/s and an air pressure of approx. 4 bar. The inner geometry of the cubic-shaped scaffolds was adjusted as follows: 3 layers with a strand-to-strand distance of 0.3 mm and a further 36 layers with a strand-to-strand distance as follows: 0.43 mm (Scaffold A), 0.56 mm (Scaffold B), 0.69 mm (Scaffold C), 0.82 mm (Scaffold D), 0.95 mm (Scaffold E) or 1.08 mm (Scaffold F). Layer-to-layer orientation was 90° or 45° in relation to the scaffolds’ edges. To test the stability, individualized scaffolds were plotted using the same technique. The inner geometry of the individualized scaffolds was adjusted as follows: the drilling axis was aligned parallel to the direction of fabrication (*Z*-axis). The filling pattern was then set at −45°/45°. After plotting, scaffolds were incubated for 72 h in a water-saturated atmosphere (humidity 95%, temperature 37 °C), followed by three intensive washing steps in acetone to remove residual oil from the CPC paste. Afterwards, the scaffolds were dried under a fume cupboard. Bio-Oss^®^Blocks (Geistlich Biomaterials Vertriebsgesellschaft mbH, Baden-Baden, Germany) were used as a control group. The blocks were bisected into 10 × 10 × 10 mm cubes for Zwick testing and colonization.

### 2.3. Scaffold Characterization

The shape and macro porosity of the printed scaffolds were initially studied by stereo microscopic investigation using a Leica M205C equipped with a DFC295 camera (Leica, Wetzlar, Germay). Scanning electron microscopy (SEM) was performed to assess the microporosity and colonization of the scaffolds. For this purpose, the samples were coated with gold using a Cressington Sputter Coater 108 auto (Crawley, UK). The following process sputtering parameters were applied: *p* = 0.1 mbar, I = 30 mA and a target-sample surface distance of 55–60 mm. Surface morphology and cell colonization were imaged with a Philips XL 30 ESEM scanning electron microscope (Philips Electron optics GmbH, Kassel, Germany) utilizing an SE detector. For the image acquisitions, depending on the material and imaging type (overview or detail), the voltage varied from 10 kV to 20 kV and the working distance varied from 4.5 to 20 mm. The mechanical characterization was performed via a uniaxial compressive test with a speed of 100 NM in the vertical direction by using a Zwick universal testing machine (Z010 equipped with a 10 kN load cell; Zwick, Ulm, Germany). Compressive modulus and compressive strength were calculated from the obtained data (*n* = 5) and representative curves are shown.

### 2.4. Colonization

After 24 h of re-equilibration to culture conditions in DMEM, scaffolds were seeded with hMSC at a density of 1 × 106 cells per scaffold, in order to study colonization. Therefore, cells were expanded in DMEM containing 15% fetal calf serum (FCS), 1% L-glutamate and 1% Pen/Strep (all from GIBCO, Germany). 

For seeding, the immersed scaffolds were placed into 5 mL tubes and a 5000 µL cell suspension containing 1 × 106 cells. Scaffold colonization was performed by way of a rotation method over a period of 6 h, as previously described by Korn et al. [24]. Tubes were rotated every 30 min by 540°, while being stored at 37 °C and 5% CO_2_. Finally, the scaffolds were placed in 24-well plates, covered with culture medium and incubated for up to 12 weeks. The medium was replaced twice a week. Live/Dead staining was performed by using a Live/Dead Cell Staining Kit II (Promocell, Germany) according to the manufacturer´s instructions. For fluorescence microscopic analyses, colonized scaffolds were fixed using 4% formaldehyde. Actin cytoskeletons and cell nuclei were stained with AlexaFlour 488^®^ phalloidin (Invitrogen, Waltham, MA, USA) and DAPI (Sigma Aldrich, Taufkirchen, Germany). All microscopic investigations were performed with a Keyence BZ9000E (Keyence, Neu-Isenburg, Germany). For the determination of cell number and LDH activity, frozen samples were thawed, followed by cell lysis with PBS containing 1% Triton X-100. During cell lysis, each sample was sonicated for 1 min at 80 W. One aliquot of the cell suspension was used to determine LDH activity via Cytotox96 kit (Promega, Madison, WI, USA) according to the manufacturer´s instructions. The LDH activity was correlated with the cell number using a calibration curve. Total DNA was quantified for the calculation of cell number using a calibration curve of cells. Therefore, DNA was quantified via Quantifluor assay (Promega, Madison, WI, USA) according to the manufacturer´s instructions. All measurements were performed by using a spectrofluorometer (Infinite M200pro; Tecan Trading AG, Männedorf, Switzerland). 

### 2.5. Statistics

For statistical analyses, GraphPad Prism 6.0 software (San Diego, CA, USA) was used. All experiments were performed at defined time points using replicates as indicated in the figure captions. The results were expressed as mean ± standard deviation (SD). One-way analysis of variance (ANOVA) with Bonferroni adjustment of *p*-values was performed to analyze statistical significance. Therefore, *p* ≤ 0.05 was considered to indicate statistical significance.

## 3. Results

### 3.1. Scaffold Fabrication and Mechanical Testing

**Figure 1:**
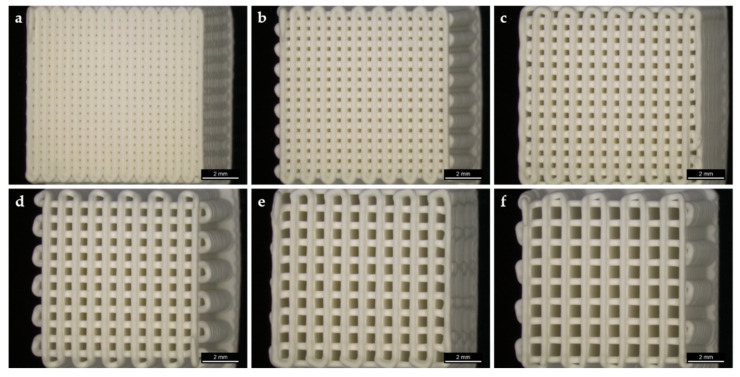
Three dimensional (3D) plotted scaffolds with different pore sizes. Strand-to-strand-distance µm/pore size µm: (**a**) 430/100; (**b**) 560/230; (**c**) 690/360; (**d**) 820/490; (**e**) 950/620; (**f**) 1080/750. Scale bars: 2 mm.

Cube shaped scaffolds with six different strand distances, resulting in six different macro porosities, possessed a well-defined porous structure (Figure 1). 

**Figure 2:**
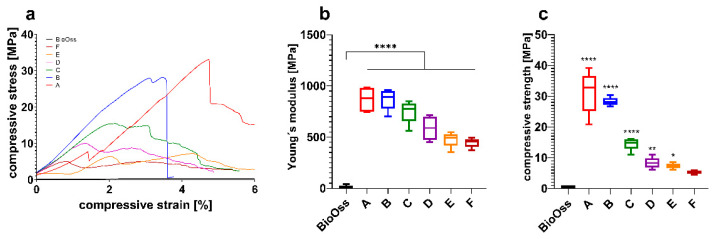
Mechanical properties of CPC scaffolds A–F (see also Table 1) with different pore sizes in comparison to BioOss^®^. (**a**) Representative compressive stress–strain curves. (**b**) Young’s modulus and (**c**) compressive strength determined from the curves (* *p* ≤ 0.05, ** *p* ≤ 0.01, **** *p* ≤ 0.0001, mean ± standard deviation, n = 5).

Comparing the CPC scaffolds, significant differences in the mechanical properties were observed (Figure 2). Data are shown in Table 1. 

The results showed that the energy absorption of the different scaffold types is greatly reduced with increasing pore size (Table 1). This intense decrease is also shown in the compressive strength data (Figure 2c). Nevertheless, all investigated scaffolds, independently from pore size, showed a higher energy absorption and strength in comparison to the control group (BioOss^®^).

### 3.2. Colonization of Scaffolds

**Figure 3:**
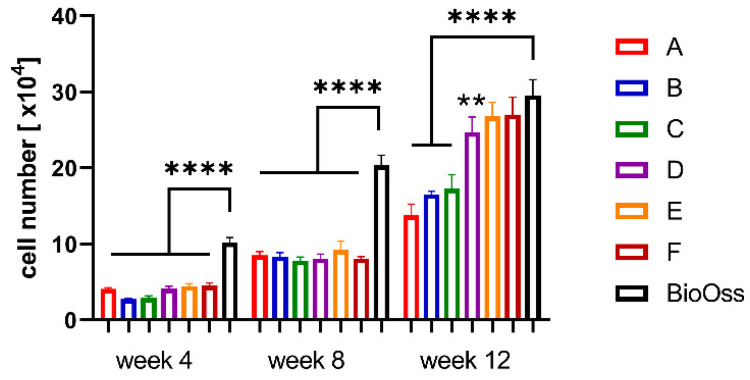
Proliferation of MSCs on/in the scaffolds depending on the various porosities after 4, 8 and 12 weeks in comparison to BioOss^®^ (strand-to-strand-distance µm/pore size µm: A: 430/100; B: 560/230; C: 690/360; D: 820/490; E: 950/620; F: 1080/750; ** *p* ≤ 0.01, **** *p* ≤ 0.0001, mean ± standard deviation, n = 5).

In order to enable colonization within porous scaffolds, it is important to equilibrate the scaffolds for 24 h in a cell culture medium before seeding. Following the equilibration, the scaffolds were seeded with hMSCs and incubated for up to 12 weeks in order to study colonization. Determination of DNA and LDH was performed to evaluate the proliferation of cells cultivated on the scaffolds (Figure 3). After four weeks, cells completely covered the CPC strands of the topmost layer, but the cell number of the CPC scaffolds was significantly lower in comparison to the control (BioOss^®^). After 12 weeks, the cell number of scaffolds A, B and C was significantly lower in comparison to the control, while scaffolds D, E and F showed a colonization comparable to that of BioOss^®^.

**Figure 4:**
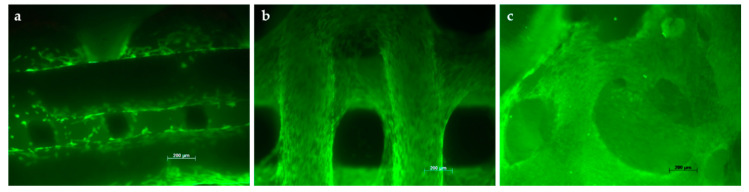
Colonization of cubic scaffolds with MSCs: Scaffold B (**a**), Scaffold D (**b**) and BioOss^®^ (**c**) after 28 days. (Live/Dead-staining).

Live/Dead staining was carried out to assess the viability of the cells. Through the culture period, the density of living cells (stained green) increased. Furthermore, microscopically, a widespread colonization of scaffolds was observed earlier in those with a higher pore size in comparison to those with a smaller pore size. The cells covered the superficial cement strands and also those in subjacent layers. Scaffold D, with a strand-to strand-distance of 820 µm and a pore size of 490 µm, exhibited a colonization similar to that of the control (BioOss^®^) (Figure 4). In all cases, no dead cells (stained red) were detected.

**Figure 5:**
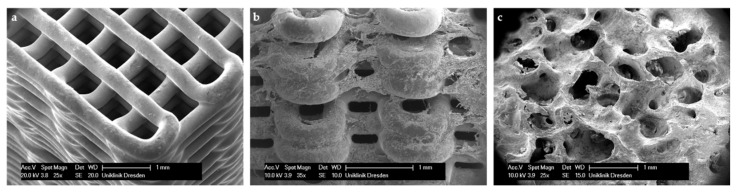
SEM imaging of CPC scaffold D non-colonized (**a**) and after 28 days (**b**) in comparison to BioOss^®^ (**c**).

Microscopic SEM evaluation of colonized scaffolds after 28 days revealed that, similar to the Life/Dead staining, cells completely covered the scaffold strands. For Scaffold D especially, cell clusters bridging the interspaces between strands were observed (Figure 5).

### 3.3. Scaffold Design for Intraoral Applications

**Figure 6:**
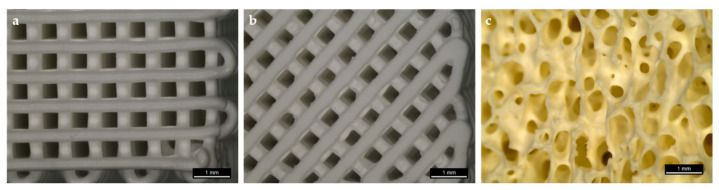
Three dimensional (3D) plotted scaffold with defined pore size (0.49 mm) and strand distance (0.82 mm): (**a**) strand orientation 90° related to edge, (**b**) strand orientation 45° related to edge, (**c**) BioOss^®^; scale bars: 1 mm.

In addition to the above mentioned scaffolds with strands laying 90° in relation to the scaffold’s edges, we printed scaffolds with strands laying 45° related to the scaffold’s edges to enhance the surface area (Figure 6). 

**Figure 7:**
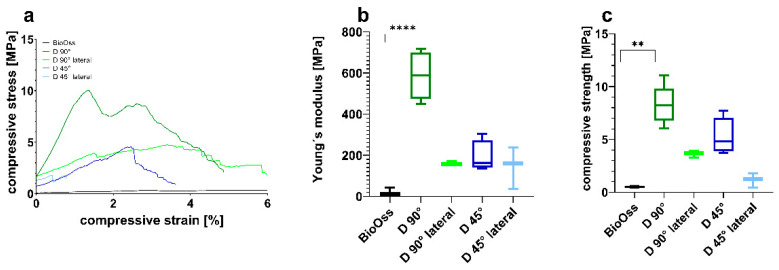
Mechanical properties of CPC scaffolds with different strand orientations in comparison with BioOss^®^. (**a**) Representative compressive stress–strain curves. (**b**) Young’s modulus and (**c**) compressive strength determined from the curves (** *p* ≤ 0.01, **** *p* ≤ 0.0001, mean ± standard deviation, n = 5).

For this purpose, we used a pore size of 0.49 mm and a strand-to-strand-distance of 0.82 mm. Since the chewing process causes the application of forces from different directions, we investigated both strand orientations applying uniaxial strength from above and laterally. Young’s modulus (Figure 7b) was estimated from the initial slope of the stress–strain curves (Figure 7a) in the elastic region. Compressive strength (Figure 7c) was evaluated from the stress–strain curves (Figure 7a). Data are presented in Table 2.

The results have shown that the energy absorption of the different scaffold types varies not only depending on strand orientation but also on the direction that the strength is applied from. Scaffolds with a 90° strand orientation seem to be more stable compared to 45° scaffolds (Figure 7c). 

It also seems as if the compressive strength resistance of both strand orientations is significantly lower when the strength is applied laterally. Nevertheless, all investigated scaffolds have shown a higher energy absorption in comparison with the control group (BioOss^®^). 

### 3.4. Preliminary Investigations of Individual Scaffolds for Clinical Cases

**Figure 8:**
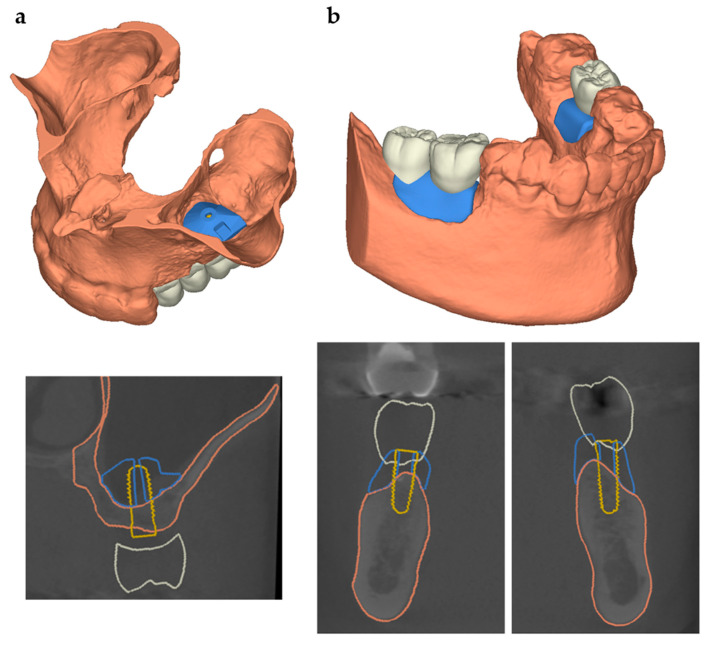
Digital backward planning based on CBCT data. (**a**) Sinus floor elevation in region 26, (**b**) bilateral onlay graft in the posterior mandible (white: prosthetic restoration; yellow: dental implant; orange: bone contour; blue: planned CPC scaffold.

For the manufacturing of patient-specific scaffolds in order to reconstruct individual bone defects, a high-resolution model of the defect site is necessary. Based on patients’ computed tomography data, irregular shaped macroporous scaffolds were designed in close collaboration with maxillofacial surgeons via digital backward planning. For this study, we analyzed different clinical cases—sinus floor elevation and onlay osteoplasty—in different regions, shapes and sizes (Figure 8).

**Figure 9:**
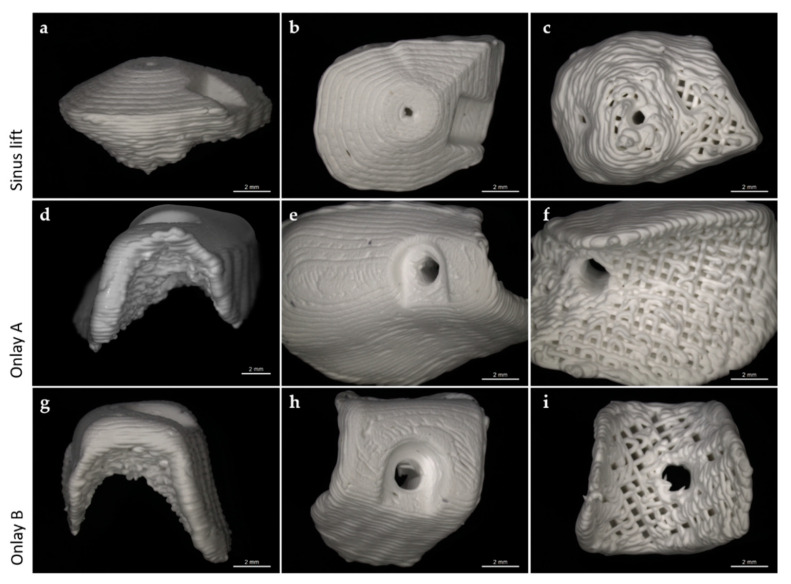
Individual scaffold for sinus floor elevation (**a**–**c**) and two onlay grafts in the lower jaw (region 36, 46: (**d**–**f**); region 47: (**g**–**i**)).

According to the anatomical requirements of the defect site, the digital planned scaffolds were printed by INNOTERE GmbH (Radebeul, Germany). Since blood and bone cells are expected to migrate into the scaffold from the prepared adjacent bone, the bone-facing part of the scaffolds was printed with the approved pore size of 0.49 mm and a strand distance of 0.82 mm. To avoid fibroblasts or keratinocytes infiltrating the scaffold from the soft tissue facing side, this part was printed with a strand distance of 0.3 mm (Figure 9) to achieve a similar effect to that of using the membrane technique. 

The mechanical properties of the individual scaffolds were tested by conducting uniaxial compression tests. Data are shown in Table 3. 

**Figure 10:**
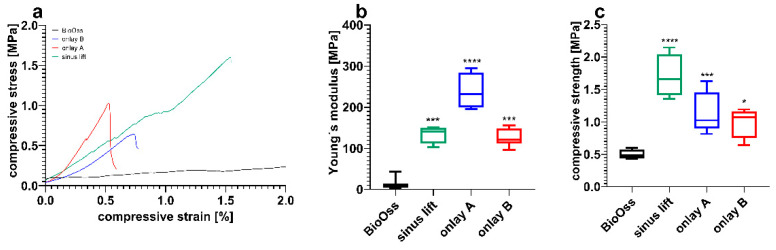
Mechanical properties of individual scaffolds in comparison with BioOss^®^. (**a**) Representative compressive stress–strain curves. (**b**) Young’s modulus and (**c**) compressive strength determined from the curves (* *p* ≤ 0.05, *** *p* ≤ 0.001, **** *p* ≤ 0.0001, mean ± standard deviation, n = 5).

Young’s modulus (Figure 10b) was estimated from the initial slope of stress–strain curves (Figure 10a) in the elastic region. Compressive strength (Figure 10c) was evaluated from stress–strain curves (Figure 10a). The results showed that the energy absorptions of the sinus lift scaffold and onlay B are comparable, while onlay A showed a higher energy absorption. Nevertheless, all investigated scaffolds, independently from their shape, showed a higher energy absorption and strength in comparison with the control group (BioOss^®^).

## 4. Discussion

This study aimed to determine the optimal porosity of CPC scaffolds for bone augmentation in maxillofacial surgery according to specific indications. Regarding stability and cell infiltration, the data presented suggest that pore sizes of 750 µm allow for a significantly higher increase in cell colonization compared to smaller pores after 12 weeks (see Figure 3). Furthermore, the stability of the CPC cubes increases up to a pore size of 100 µm with an observed compressive strength of 31.3 ± 6.8 MPa and a Young’s Modulus of 870 ± 117 MPa (see Figure 2). Nevertheless, individual CPC scaffolds, which are closer to clinical conditions, have shown a much lower compressive strength resistance depending on the respective site of destination. Thus, in certain cases, the porosity of individual scaffolds in maxillofacial surgery needs to be adapted with an acceptance of the concomitant decrease of cell infiltration.

Bone augmentation is performed when bony defects compromise the function and aesthetics of the orofacial system [25,26,27,28]. One of its main functions is to grind food as the first step of digestion. This is conducted by frequently repeated contraction of the chewing muscles. The chewing muscles belong to the strongest muscles in the human body. During the chewing process, forces beyond 200 N emerge depending on the region within the oral cavity [20,21]. The highest pressure can be measured in the lateral region of the jaws since this is the chewing center [20]. In other sites, such as the sinus floor, the anterior parts of the jaws or parts of the facial bone, the pressure is much lower [20]. Assuming a full dentition with an average chewing surface of approx. 6 cm^2^, this corresponds to a pressure of approx. 0.4 MPa per tooth.

By Zwick universal testing, uniaxial compression can be applied to the test object. This makes it an appropriate testing procedure to resemble the in vivo situation, as teeth and the adjacent bone are stressed in a similar way. By testing standardized CPC cubes, 32 MPa was measured as the highest compressive strength withstood by scaffold A, which had a strand-to-strand distance of 430 µm and a pore size of 100 µm. This easily exceeds the essential requirements (0.4 MPa) for an in vivo application. Compared to the control group (BioOss^®^), which is commonly used for bone substitution, the applicable compressive strength of scaffold A was 60 times higher. 

Nevertheless, the colonization experiments have shown that pore sizes of 100 µm (scaffold A) are too small to let cells quickly migrate into the scaffold. Due to the small pores, instead of infiltrating, cells instead colonized the scaffold’s outer surface. 

**Figure 11:**
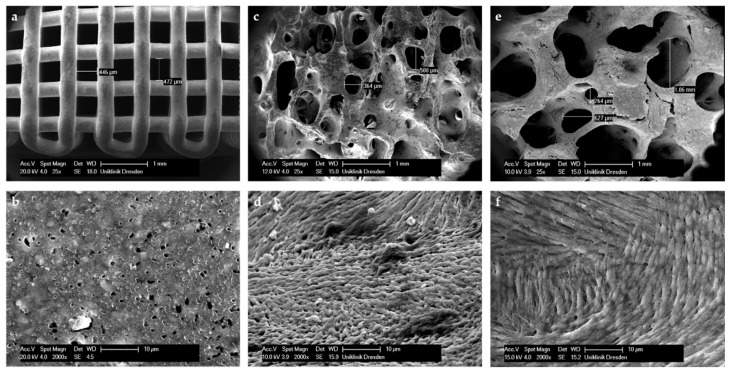
SEM imaging of CPC scaffold D (**a**,**b**), BioOss ^®^ (**c**,**d**) and natural bone (**e**,**f**).

As depicted in Figure 3, in our case, a porosity of 750 µm (scaffold F) seems to be the best for cell infiltration. Nevertheless, cell numbers observed in the control group (BioOss^®^) were still superior from week 4 onwards. The reason for this could be the surface of BioOss^®^, which mimics the surface of natural bone better than CPC does (Figure 11). As seen in Figure 11, the CPC’s surface is smooth, whereas the surfaces of BioOss^®^ and natural bone have many micro irregularities. These irregularities lead to an enhanced attachment area for cells. 

However, this advantage seems to decrease over time. Up to week 12, the difference in cell numbers between BioOss^®^ and the CPC scaffolds with high porosity decreases continuously until they are nearly the same after week 12 (Figure 3). The influence of surface roughness on cell adhesion and function has been discussed in several studies [29,30]. The observed micro irregularities not only offer more surface for cell binding, but they also strengthen the adsorption of proteins and the extracellular matrix, which enhances the cells’ adhesion and function. This effect was observed for different biomaterials and cells [31,32,33]. To improve the early cell adhesion on CPC scaffolds, the CPC could be enriched with nanoparticles such as bioactive glass, as shown by Richter et al. [34]. Thereby, the CPC’s surface could be enriched with irregularities to better resemble natural bone.

In contrast, due to a decreased surface and increased strand-to-strand distance, a higher porosity goes along with a significant decrease of the compressive strength resistance and Young’s Modulus. As depicted in Figure 2, scaffold F (pore size 750 µm) shows a compressive strength of 5.2 ± 0.6 MPa, which is much higher compared to that of the control group (0.5 ± 0.007 MPa). In Figure 5, it is shown that the porosity of scaffold D seems to be similar to that of BioOss^®^. Nevertheless, scaffold D is much more stable. This superior stability of the CPC scaffolds compared with BioOss^®^ may be caused by the differences in their architecture. Microscopically, a natural spongious bone, similar to the architecture of BioOss^®^, can be observed. As shown in Figure 6, the spongious trabeculae are arranged irregularly in contrast to the strands of the CPC scaffolds. This regular arrangement of the CPC strands may be the reason for the higher compressive strength resistance. The pressure can be evenly deviated above the whole surface. 

The Young’s Modulus of the observed CPC cubes ranged from 444 ± 44 MPa to 870 ± 117 MPa. Human bone has a Young’s Modulus of about 4.42 MPa as shown by Boughton et al. [35]. It is worth noting that Boughton et al. investigated cortical bone samples from femoral necks, the mechanical properties of which may differ from jaw and facial bone. Furthermore, the donors from which the bone was harvested had a mean age of 69 years. Due to the fact that age and chronic diseases have a significant impact on bone density, architecture and mechanical properties [36], these values may not be comparable to the jaw bones of patients undergoing maxillofacial surgery. 

Nevertheless, the Young’s Modulus of natural human bone seems to be much lower in comparison to the observed CPC scaffolds. The orofacial system is in permanent motion and underlies continuous dynamics. In such dynamic systems, differences of the Young’s modulus can be crucial. They could lead to micro movements between scaffold and bone and thereby compromise the scaffold’s integration. Here, BioOss^®^ is much closer to natural bone, due to its natural origin and closely mimics natural bone tissue. In contrast to this, the CPC scaffolds consist of artificial tri-calcium phosphate and are manufactured by using amorphous paste. This may be why they are more brittle and less elastic. Besides the differences in the Young’s Modulus, a high brittleness could compromise the intraoperative handling since the scaffolds have to be fixed with titanium screws. If the scaffolds are too brittle, they may break when the screw is inserted. This could probably be avoided by integrating a screw channel preliminarily and thereby decreasing the stress in the CPC while inserting the screw (as shown in Figure 9). Nevertheless, a pore size of 750 µm seems to be adequate for facilitating a high infiltration of cells and still meeting the mechanical requirements in the orofacial system. These findings were, however, observed in regularly shaped, cubic CPC scaffolds.

Knowing that this design might fail in simulating in situ settings with complex shaped defects, we additionally investigated clinical cases. Three cases were selected that displayed typical intraoral regions with mechanical requirements different to those of a bone scaffold (Figure 8): sinus floor elevation, onlay osteoplasty located posterior to the remaining teeth (onlay A) and onlay osteoplasty embraced by remaining teeth (onlay B). 

Sinus floor elevation is a procedure that is used to create a sufficient base for dental implants in the posterior maxilla [37,38,39]. This is realized by inserting the bone substitution material through a bony window that has to be cut into the lateral wall of the maxillary sinus. During the healing period prior to implant insertion, it is not affected by pressure or movement. Due to these highly protected conditions during the healing period, a CPC scaffold for sinus floor elevation does not need to withstand a high compressive strength. Therefore, in such cases it could be advantageous to choose large pore sizes to gain the maximum cell infiltration. According to our findings, 750 µm would be the appropriate pore size in this case. Nevertheless, due to its pyramidal and compact geometry, the scaffold reaches high compressive strength resistance (1.7 ± 0.3 MPa) and therefore exceeds the essential requirements of the maxillary sinus. Considering this, even larger pore sizes could be assumed for such cases. The control group also seems to be a good choice for sinus floor elevation. As discussed above, the low compressive strength resistance of BioOss^®^ can be neglected. According to Figure 3, BioOss^®^ would even allow for a faster and larger increase of cell colonization on its surface compared to CPC scaffolds. This advantage of BioOss^®^ could probably be compensated for by coating the CPC scaffolds with collagen as shown by Lee et al. [40]. Moreover, there are several advantages that favor CPC scaffolds. In contrast to BioOss^®^, CPC scaffolds can be individually designed based on a CBCT scan. Patient-specific geometries can be printed [24,41,42], thus they will fit perfectly to the defect site. The surgeon saves time during surgery since there is no need to prepare or adapt the scaffold intraoperatively. The planning of the augmentation is conducted before the surgery, which minimizes the risk of over- or under treatment. Additionally, CPC scaffolds can be printed with a graded porosity. Thus, the outer “soft tissue facing side” of the scaffold can be printed densely so that fibroblasts are not able to immigrate. Usually for this purpose additional membranes need to be placed to cover the defect site [43,44,45]. These membranes always come with the risk of early dehiscences and inflammation [46]. To summarize, for sinus floor elevation, CPC scaffolds with a pore size of 750 µm seem to be a sufficient tool.

Onlay osteoplasty in combination with dental implants is a standard procedure for the functional rehabilitation of highly atrophic jaws [27]. For onlay osteoplasty, the surgeon prepares a mucoperiosteal flap and fixes the bone substitution material directly to the defect site. In these cases, the bone scaffold is located submucosally. Hence, it is exposed to motions and forces directly after surgery. In this study, we simulated two clinical cases: region of teeth 46 and 47 (onlay A: Figure 9D–F) and region 36 (onlay B: Figure 10G–I). Both sites are under permanent pressure due to their location in the chewing center. Remarkably, there is an important difference between both cases. Onlay A covers an area of two teeth and there are no teeth posterior to the defect. Therefore, it needs to hold those forces that emerge in the chewing center on its own. In contrast, onlay B covers the area of one tooth and is embraced by teeth, anteriorly and posteriorly. The adjacent teeth may protect the scaffold from high compressive strength. Nevertheless, both scaffolds need to resist a higher compressive strength compared to that resisted by the sinus floor scaffold. Zwick testing of onlay A and onlay B revealed that the favored pore size of 750 µm is not stable enough to withstand the forces during the chewing process. The same effect was shown with a pore size of 620 µm (data not shown). 

The pore size that was found to be strong enough to withstand the forces in the chewing center and also showed excellent colonization data was 490 µm with a compressive strength resistance of at least 1.0 ± 0.2 MPa. The individual scaffolds with pores of 490 µm have a Young’s Modulus of 239 ± 45 MPa and 127 ± 22 MPa for onlay A and onlay B, respectively. As mentioned above, the Young’s Modulus of human bone is approx. 4.42 MPa [35]. The mandible, especially, is known to be flexible and moved by different surrounding muscles. Unfortunately, the mechanical testing has shown that even very wide pore sizes, such as 750 µm, cannot affect the Young’s Modulus to the extent that it would be comparable to human bone (Figure 2B). Nevertheless, CPC scaffolds with pore sizes of 490 µm seem to be a solid option for onlay osteoplasty in the lower lateral jaw. The superior compressive strength resistance compared to the control group especially makes CPC scaffolds an appropriate alternative to autologous bone, which is mostly used for onlay osteoplasty. Nevertheless, this study has certain limitations. The outer soft tissue facing layer was printed as densely as possible to prevent the migration of mucosal cells. Our experimental setting does not clarify whether our scaffold design fulfills this requirement properly. Furthermore, there are various other patient specific aspects that influence the integration of the scaffolds, such as certain comorbidities or lifestyle habits. Moreover, the degradation time of scaffolds is of high clinical relevance and it could be hypothesized that pore size also affects degradation time. To answer this question, an in vivo study would have to be conducted.

## 5. Conclusions

Our findings suggest that the pore size of CPC scaffolds for bone augmentation in maxillofacial surgery should be adapted for the planned site. CPC scaffolds for augmentation sites that are not exposed to high forces, such as the sinus floor, could be printed with a pore size of 750 µm to benefit from the enhanced cell infiltration. In contrast, CPC scaffolds for bone augmentation in areas exposed to high pressures, such as the lateral mandible, should be planned with a pore size of 490 µm. This pore size facilitates adequate cell infiltration and simultaneously meets the mechanical requirements in these highly stressed areas. 

## Figures and Tables

**Table 1:** Mechanical properties of 3D printed scaffolds A–F with different strand-to-strand distances and pore sizes and control.

Scaffold	Strand-to-Strand- Distance (µm)	Pore Size (µm)	Young’s Modulus # (MPa)	Compressive Strength # (MPa)
A	430	100	870 ± 117	31.3 ± 6.8
B	560	230	870 ± 101	28.3 ± 1.3
C	690	360	749 ± 110	14.5 ± 2.0
D	820	490	586 ± 118	8.3 ± 1.8
E	950	620	477 ± 73	7.4 ± 0.9
F	1080	750	444 ± 44	5.2 ± 0.6
Control			7 ± 4	0.5 ± 0.007

# mean ± standard deviation (SD), n = 5.

**Table 2:** Mechanical properties of scaffolds with different printing directions in comparison to the control.

Scaffold	Young’s Modulus # (MPa)	Compressive Strength Resistance # (MPa)
90°	586 ± 118	8.3 ± 1.8
90° lateral	159 ± 11	3.6 ± 0.3
45°	191 ± 77	5.3 ± 1.7
45° lateral	145 ± 101	1.2 ± 0.7
Control	7 ± 4	0.5 ± 0.007

# mean ± standard deviation (SD).

**Table 3:** Mechanical properties of patient individual scaffolds and control group.

Scaffold	Young’s Modulus # (MPa)	Compressive Strength Resistance # (MPa)
Sinus lift	135 ± 21	1.7 ± 0.3
Onlay A	239 ± 45	1.2 ± 0.3
Onlay B	127 ± 22	1.0 ± 0.2
Control	7 ± 4	0.5 ± 0.007

# mean ± standard deviation (SD).

## Data Availability

The data presented in this study are available on request from the corresponding author. The data are not publicly available due to privacy restrictions.

## References

[1] Shamsoddin E., Houshmand B., Golabgiran M. (2019). Biomaterial selection for bone augmentation in implant dentistry: A systematic review. J. Adv. Pharm. Technol. Res..

[2] Starch-Jensen T., Deluiz D., Deb S., Bruun N.H., Tinoco E.M.B. (2020). Harvesting of Autogenous Bone Graft from the Ascending Mandibular Ramus Compared with the Chin Region: A Systematic Review and Meta-Analysis Focusing on Complications and Donor Site Morbidity. J. Oral Maxillofac. Res..

[3] Scheerlinck L.M., Muradin M.S., van der Bilt A., Meijer G.J., Koole R., Van Cann E.M. (2013). Donor site complications in bone grafting: Comparison of iliac crest, calvarial, and mandibular ramus bone. Int. J. Oral Maxillofac. Implant..

[4] Saha A., Shah S., Waknis P., Bhujbal P., Aher S., Vaswani V. (2019). Comparison of minimally invasive versus conventional open harvesting technique for iliac bone graft in secondary alveolar bone grafting in cleft palate patients: A systematic review. J. Korean Assoc. Oral Maxillofac. Surg..

[5] Jakoi A.M., Iorio J.A., Cahill P.J. (2015). Autologous bone graft harvesting: A review of grafts and surgical techniques. Musculoskelet. Surg..

[6] Mishra A., Srivastava V. (2021). Biomaterials and 3D printing techniques used in the medical field. J. Med. Eng. Technol..

[7] Aimar A., Palermo A., Innocenti B. (2019). The Role of 3D Printing in Medical Applications: A State of the Art. J. Healthc. Eng..

[8] Parmar H., Khan T., Tucci F., Umer R., Carlone P. (2021). Advanced robotics and additive manufacturing of composites: Towards a new era in Industry 4.0. Mater. Manuf. Process..

[9] Öztürkmen Y., Canikliołu M., Karamehmetołu M., Fiükür E. (2010). Calcium phosphate cement augmentation in the treatment of depressed tibial plateau fractures with open reduction and internal fixation. Acta. Orthop. Et. Traumatol. Turc..

[10] Ji C., Ahn J.G. (2010). Clinical experience of the brushite calcium phosphate cement for the repair and augmentation of surgically induced cranial defects following the pterional craniotomy. J. Korean Neurosurg. Soc..

[11] Xu H.H.K., Wang P., Wang L., Bao C., Chen Q., Weir M.D., Chow L.C., Zhao L., Zhou X., Reynolds M.A. (2017). Calcium phosphate cements for bone engineering and their biological properties. Bone Res..

[12] Reitmaier S., Kovtun A., Schuelke J., Kanter B., Lemm M., Hoess A., Heinemann S., Nies B., Ignatius A. (2018). Strontium(II) and mechanical loading additively augment bone formation in calcium phosphate scaffolds. J. Orthop. Res..

[13] Cha J.K., Kim C., Pae H.C., Lee J.S., Jung U.W., Choi S.H. (2019). Maxillary sinus augmentation using biphasic calcium phosphate: Dimensional stability results after 3-6 years. J. Periodontal. Implant. Sci..

[14] Wach T., Kozakiewicz M. (2020). Fast-versus slow-resorbable calcium phosphate bone substitute materials-texture analysis after 12 months of observation. Materials.

[15] Marongiu G., Verona M., Cardoni G., Capone A. (2020). Synthetic bone substitutes and mechanical devices for the augmentation of osteoporotic proximal humeral fractures: A systematic review of clinical studies. J. Funct. Biomater..

[16] Rolvien T., Barbeck M., Wenisch S., Amling M., Krause M. (2018). Cellular Mechanisms Responsible for Success and Failure of Bone Substitute Materials. Int. J. Mol. Sci..

[17] Pepelassi E., Perrea D., Dontas I., Ulm C., Vrotsos I., Tangl S. (2019). Porous Titanium Granules in comparison with Autogenous Bone Graft in Femoral Osseous Defects: A Histomorphometric Study of Bone Regeneration and Osseointegration in Rabbits. Biomed. Res. Int..

[18] Duda M., Pajak J. (2004). The issue of bioresorption of the Bio-Oss xenogeneic bone substitute in bone defects. Ann. Univ. Mariae Curie-Skłodowska. Sect. D Med..

[19] Schlegel A.K., Donath K. (1998). BIO-OSS®-A resorbable bone substitute?. J. Long-Term Eff. Med. Implant..

[20] Ledogar J.A., Dechow P.C., Wang Q., Gharpure P.H., Gordon A.D., Baab K.L., Smith A.L., Weber G.W., Grosse I.R., Ross C.F. (2016). Human feeding biomechanics: Performance, variation, and functional constraints. PeerJ.

[21] Righetti M.A., Taube O.L.S., Palinkas M., Gonçalves L.M.N., Esposto D.S., de Mello E.C., Regalo I.H., Regalo S.C.H., Siéssere S. (2020). Osteoarthrosis: Analyze of the Molar Bite Force, Thickness and Masticatory Efficiency. Prague Med. Rep..

[22] La Monaca G., Iezzi G., Cristalli M.P., Pranno N., Sfasciotti G.L., Vozza I. (2018). Comparative Histological and Histomorphometric Results of Six Biomaterials Used in Two-Stage Maxillary Sinus Augmentation Model after 6-Month Healing. Biomed. Res. Int..

[23] Yamada M., Egusa H. (2018). Current bone substitutes for implant dentistry. J. Prosthodont Res..

[24] Korn P., Ahlfeld T., Lahmeyer F., Kilian D., Sembdner P., Stelzer R., Pradel W., Franke A., Rauner M., Range U. (2020). 3D Printing of Bone Grafts for Cleft Alveolar Osteoplasty-In vivo Evaluation in a Preclinical Model. Front. Bioeng. Biotechnol..

[25] Sakkas A., Wilde F., Heufelder M., Winter K., Schramm A. (2017). Autogenous bone grafts in oral implantology—is it still a “gold standard”? A consecutive review of 279 patients with 456 clinical procedures. Int. J. Implant. Dent..

[26] Toledano-Serrabona J., Sánchez-Garcés M.Á., Sánchez-Torres A., Gay-Escoda C. (2019). Alveolar distraction osteogenesis for dental implant treatments of the vertical bone atrophy: A systematic review. Med. Oral Patol. Oral Cir. Bucal..

[27] Nguyen T.T.H., Eo M.Y., Kuk T.S., Myoung H., Kim S.M. (2019). Rehabilitation of atrophic jaw using iliac onlay bone graft combined with dental implants. Int. J. Implant. Dent..

[28] Salmen F.S., Oliveira M.R., Gabrielli M.A.C., Piveta A.C.G., Pereira Filho V.A., Ganrielli M.F.R. (2017). Bone grafting for alveolar ridge reconstruction. Review of 166 cases. Rev. Do Colégio Bras. De Cir..

[29] Stepanovska J., Matejka R., Rosina J., Bacakova L., Kolarova H. (2020). Treatments for enhancing the biocompatibility of titanium implants. Biomed. Pap..

[30] Samavedi S., Whittington A.R., Goldstein A.S. (2013). Calcium phosphate ceramics in bone tissue engineering: A review of properties and their influence on cell behavior. Acta Biomater..

[31] Hu X., Mei S., Wang F., Qian J., Xie D., Zhao J., Yang L., Wu Z., Wei J. (2021). Implantable PEKK/tantalum microparticles composite with improved surface performances for regulating cell behaviors, promoting bone formation and osseointegration. Bioact. Mater..

[32] Deligianni D.D., Katsala N.D., Koutsoukos P.G., Missirlis Y.F. (2000). Effect of surface roughness of hydroxyapatite on human bone marrow cell adhesion, proliferation, differentiation and detachment strength. Biomaterials.

[33] Zhou K., Li Y., Zhang L., Jin L., Yuan F., Tan J., Yuan G., Pei J. (2021). Nano-micrometer surface roughness gradients reveal topographical influences on differentiating responses of vascular cells on biodegradable magnesium. Bioact. Mater..

[34] Richter R.F., Ahlfeld T., Gelinsky M., Lode A. (2019). Development and characterization of composites consisting of calcium phosphate cements and mesoporous bioactive glass for extrusion-based fabrication. Materials.

[35] Boughton O.R., Ma S., Zhao S., Arnold M., Lewis A., Hansen U., Cobb J.P., Giuliani F., Abel R.L. (2018). Measuring bone stiffness using spherical indentation. PLoS ONE.

[36] Oftadeh R., Perez-Viloria M., Villa-Camacho J.C., Vaziri A., Nazarian A. (2015). Biomechanics and Mechanobiology of Trabecular Bone: A Review. J. Biomech. Eng..

[37] Maska B., Lin G.-H., Othman A., Behdin S., Travan S., Benavides E., Kapila Y. (2017). Dental implants and grafting success remain high despite large variations in maxillary sinus mucosal thickening. Int. J. Implant. Dent..

[38] Starch-Jensen T., Jensen J.D. (2017). Maxillary Sinus Floor Augmentation: A Review of Selected Treatment Modalities. J. Oral Maxillofac. Res..

[39] Thoma D.S., Cha J.K., Jung U.W. (2017). Treatment concepts for the posterior maxilla and mandible: Short implants versus long implants in augmented bone. J. Periodontal Implant. Sci..

[40] Lee M.H., You C., Kim K.H. (2015). Combined effect of a microporous layer and type I collagen coating on a biphasic calcium phosphate scaffold for bone tissue engineering. Materials.

[41] Ahlfeld T., Köhler T., Czichy C., Lode A., Gelinsky M. (2018). A Methylcellulose Hydrogel as Support for 3D Plotting of Complex Shaped Calcium Phosphate Scaffolds. Gels.

[42] Ahlfeld T., Akkineni A.R., Förster Y., Köhler T., Knaack S., Gelinsky M., Lode A. (2017). Design and Fabrication of Complex Scaffolds for Bone Defect Healing: Combined 3D Plotting of a Calcium Phosphate Cement and a Growth Factor-Loaded Hydrogel. Ann. Biomed. Eng..

[43] Lyu C., Shao Z., Zou D., Lu J. (2020). Ridge Alterations following Socket Preservation Using a Collagen Membrane in Dogs. BioMed Res. Int..

[44] Guarnieri R., Stefanelli L., De Angelis F., Mencio F., Pompa G., Di Carlo S. (2017). Extraction Socket Preservation Using Porcine-Derived Collagen Membrane Alone or Associated with Porcine-Derived Bone. Clinical Results of Randomized Controlled Study. J. Oral Maxillofac. Res..

[45] Kolerman R., Qahaz N., Barnea E., Mijiritsky E., Chaushu L., Tal H., Nissan J. (2020). Allograft and collagen membrane augmentation procedures preserve the bone level around implants after immediate placement and restoration. Int. J. Environ. Res. Public Health.

[46] Garcia J., Dodge A., Luepke P., Wang H.L., Kapila Y., Lin G.H. (2018). Effect of membrane exposure on guided bone regeneration: A systematic review and meta-analysis. Clin. Oral Implants Res..

